# Gender Differences in Current Received during Transcranial Electrical Stimulation

**DOI:** 10.3389/fpsyt.2014.00104

**Published:** 2014-08-15

**Authors:** Michael Russell, Theodore Goodman, Qiang Wang, Bennett Groshong, Bruce G. Lyeth

**Affiliations:** ^1^Aaken Laboratories, Davis, CA, USA; ^2^Sutter Center for Psychiatry, Sacramento, CA, USA; ^3^Center for Neuroscience, University of California Davis, Davis, CA, USA

**Keywords:** transcranial electrical stimulation, MRI, modeling, DC Stimulation, AC Stimulation

## Abstract

Low current transcranial electrical stimulation (tCS) is an effective but somewhat inconsistent tool for augmenting neuromodulation. In this study, we used 3D MRI guided electrical transcranial stimulation modeling to estimate the range of current intensities received at cortical brain tissues. Combined T1, T2, and proton density MRIs from 24 adult subjects (12 male and 12 female) were modeled with virtual electrodes placed at F3, F4, C3, and C4. Two sizes of electrodes 20 mm round and 50 mm × 45 mm were examined at 0.5, 1, and 2 mA input currents. The intensity of current received was sampled in a 1-cm sphere placed at the cortex directly under each scalp electrode. There was a 10-fold difference in the amount of current received by individuals. A large gender difference was observed with female subjects receiving significantly less current at targeted parietal cortex than male subjects when stimulated at identical current levels (*P* < 0.05). Larger electrodes delivered somewhat larger amounts of current than the smaller ones (*P* < 0.01). Electrodes in the frontal regions delivered less current than those in the parietal region (*P* < 0.05). There were large individual differences in current levels that the subjects received. Analysis of the cranial bone showed that the gender difference and the frontal parietal differences are due to differences in cranial bone. Males have more cancelous parietal bone and females more dense parietal bone (*P* < 0.01). These differences should be considered when planning tCS studies and call into question earlier reports of gender differences due to hormonal influences.

## Introduction

Low intensity transcranial electrical stimulation (tCS) is a non-invasive procedure for modifying neural networks within the brain. There is an extensive literature reporting effective treatment for a number of conditions (1–7) (and included references). However, the findings are often inconsistent with a large degree of variability between individual subjects. Earlier reports from our laboratory (8) and others (9–11) have suggested that much of the inconsistency can be accounted for by individual differences in head anatomy and differences in the amount of current received by the brain. Due to the need for more precise targeting of stimulation recent efforts have turned to modeling of current levels and pathways. Modeling current within the head has progressed steadily. Early investigators modeled current using a single-slice of MRI within the head (12), a single subject (9, 13–18), or simplified anatomic models of cranial components (13, 19, 20). Studies by Ruffini et al. (18), Miranda et al. (21), and Sadleir et al. (9) have addressed some of these issues by using MRI modeling from representative heads. Our laboratory has attempted to take modeling somewhat further by using multiple subjects and verifying the model with physical current intensity measurements to establish validity (8). We have demonstrated that it is possible to determine tissue resistance and cranial conductivity from a combination of MRI measurements (8). This MRI based technique assesses the amount of hydrogen within a tissue as an index of water and makes the assumption that the conductivity of a tissue is largely determined by the amount of salt water within the tissue. It then calculates the MRI values of hydrogen within voxels to estimate resistivity. The present report uses our previously validated MRI modeling (8) to estimate the current received at commonly used transcranial stimulation locations (22–25). We chose to constrain the electrode locations, electrode types, and stimulus intensities to produce a representative but manageable data set. Four locations were selected, one from each side of the head above the left and right parietal bones, and each side of the frontal bone. We estimated the current received at the cortex with two commonly used sizes of electrodes at three current levels. The brain region targeted by investigators is generally envisioned to be directly under the electrode site, so we modeled the current levels at the cortex directly under the electrodes.

## Materials and Methods

The subjects were 24 adults (12 male and 12 female) without known anatomical anomalies. Males had a mean age of 53 years ± 11.5 (SD) and an age range of 34–68 years. The females had a mean age of 50.5 years ± 14.3 (SD) and an age range of 21–75 years. The human subject protocol was approved by the Institutional Review Board of the Sutter Institute for Medical Research. Written informed consent was obtained from all participants.

**Figure 1:**
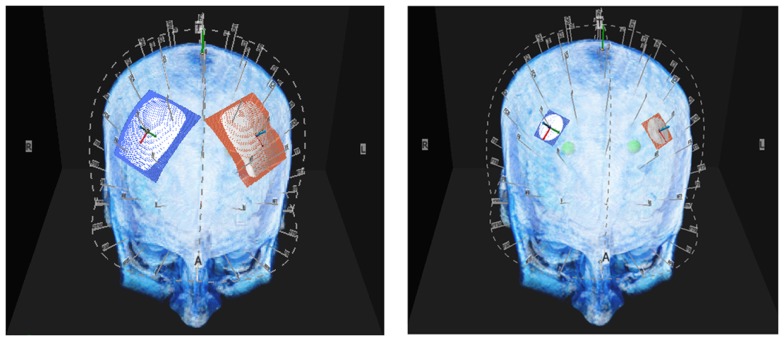
**Above are illustrations of the large and small electrodes placed on the head by the 10–10 software**. The large electrode is 50 mm × 45 mm the small electrode is 20 mm diameter.

**Figure 2:**
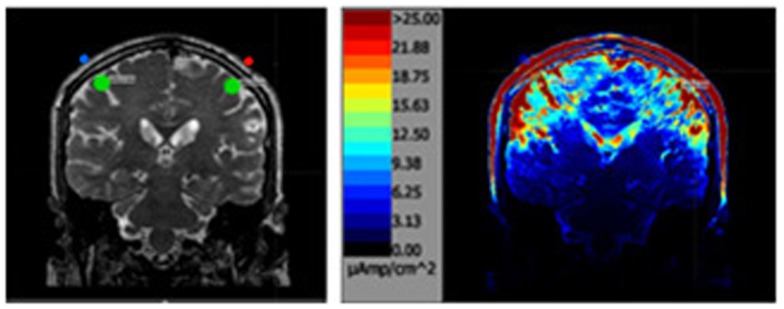
**The above left image is a T2 MRI slice with the areas of interested outlined in green directly under each parietal electrode**. The image on the right is the same slice after the simulation.

MRI scans were performed on each subject in order to model current delivered to cortical targets. Virtual electrodes were placed at four scalp location C3, C4, F3, and F4 as defined by the 10–10 system (Figure 1) and the 10–20 system (26). The modeling estimated current density delivered to a region of interest (ROI) consisting of a sphere of cortical tissue directly under the virtual electrodes (Figure 2). To encompass the range of current stimulation intensities typically used in tCS electrical current levels of 0.5, 1, and 2 mA were modeled. The current traveled in a single direction for each electrode pair C3-anode to C4-cathode and F3-anode F4-cathode. Two sizes of commonly used electrodes were modeled as 20 mm round and 50 mm × 45 mm (Figure 1). A 1 cm sphere of cortical tissue within the cranium and directly under virtual electrodes (Figure 2) was placed at four scalp locations C3, C4, F3, and F4 as defined by the 10–10 system for scalp placement.

### MRI procedures

To map the conductivity of brain tissue, a three-dimensional (3D) measurement of the hydrogen distribution in head and brain is needed rather than the typical individual slice MRI record. T1, T2, and proton density (PD) imaging each capture different aspects of the water (hydrogen) present in tissues, but each alone lacks the power to provide a precise brain conductivity model. Consequently for each subject, the data from the three types of MRI were combined to model conductivity. Diffusion weighted recordings were not used in this instance because they are considered to be unreliable at the cortex. The recordings were obtained on a three Tesla General Electric MRI (Discovery M750) machine with a 1 mm × 1 mm × 1 mm slice spacing. This combination of MRIs constitutes the Aaken Insite Protocol (27). Image data were combined into a single 3D representation and a conversion equation was applied to achieve an index of resistivity, yielding the subject’s resistivity model. The resistivity to the MRI intensity is expressed by the formula:

(1) $$R\left(v\right)=K{\left(1-v\right)}^{E}+D$$

Where *v* ε[0,1] is the normalized intensity of the combined image at the given voxel; *R* is the resistivity; *K* = 16,000; *E* = 4; and *D* = 65 are the adjustable parameters.

### Finite element analysis

Finite element and boundary conditions are given in detail at Russell et al. (8). Briefly, the subject’s resistivity distribution was translated to a rectangular prismatic linear finite element model. The model matrix equation and boundary conditions were formulated from the Galerkin equations (28). Solutions to the system matrix equations were obtained by using the conjugate gradient method (29). The finite element models solved the Laplace equation and current densities within the models were determined from the finite element model solution by calculating current densities for each voxel.

### Modeling current density

Current density maps were achieved by placing virtual electrodes [red (+) and blue (−)] according to the 10–10 system for electrode placement at four locations. Two were placed above the frontal pole F3–F4 (above frontal bone) and two above the region of the motor cortex at C3–C4 (above parietal bone). Two sizes of virtual electrodes were modeled as either 20 mm diameter round or a 50 mm × 45 mm.

The area sampled for current density was a 10 mm virtual sphere under the cranium (green circle in Figure 2) directly under each electrode. The sphere defined the voxels in the cortical region assessed for the analysis.

### Statistical analysis

Data analysis was performed using IBM SPSS software (Version 22, Chicago, IL, USA), which adheres to a general linear model. Alpha level for Type I error was set at 0.05 for rejecting null hypotheses. All data were expressed as mean ± standard error of the mean (SEM).

Differences in current density between stimulation intensity, independent of gender, location, and electrode size, was analyzed using one-way analysis of variance (ANOVA). Differences in current density between electrode size and electrode location were analyzed using repeated measures ANOVA. Differences in cancelous bone thickness were analyzed with independent *t*-test.

## Results

Table 1 presents the minimums (min), maximums (max), and means of the modeled current densities across all conditions (gender electrode placement, electrode size, and stimulation intensity in microampere per square centimeter.

**Table 1:** **The table below shows the minimum, maximum and mean values by gender and stimulus intensity for each size of electrode 50 mm by 45 square and 20 mm round**.

Gender	10–10 Local	50 mm × 45 mm square electrodes values are μA/cm^2^								
		0.5 mA			1 mA			2 mA		
		Min	Max	Mean	Min	Max	Mean	Min	Max	Mean
Male	C3	1	11.2	4.5	2	22.4	10.6	4.1	44.8	17.9
	C4	1	10.8	3.6	2	21.5	7.2	3.9	4.3	14.3
	F3	0.7	9.9	4.5	1.8	19.8	9	3.6	39.5	18
	F4	0.9	8.2	3.9	1.7	16.4	7.8	3.4	32.9	15.7
Female	C3	0.8	6.7	2.4	1.4	13.4	4.9	2.9	26.7	9.7
	C4	0.8	9	3.1	1.6	17.9	6.2	3.2	35.9	12.3
	F3	0.9	12.4	4.3	1.7	23.7	8.6	3.3	41.3	15.6
	F4	0.7	8.9	3.8	1.7	17.9	7.5	3.5	35.8	15.1

**Gender**	**10–10 Local**	**20 mm round electrodes values are μA/cm^2^**								
		**0.5 mA**			**1 mA**			**2 mA**		
		**Min**	**Max**	**Mean**	**Min**	**Max**	**Mean**	**Min**	**Max**	**Mean**

Male	C3	0.8	7.9	3.1	1.5	15.4	6.2	6.1	29.8	12.1
	C4	0.8	8.5	2.9	1.6	17.1	5.8	3.3	33.8	11.5
	F3	0.8	8	3.6	1.5	16	7.4	3	32	14.9
	F4	0.7	7.3	3.4	1.3	14.6	7.5	2.9	29.2	13.6
Female	C3	0.5	4.9	1.7	1	9.7	3.3	1.9	20.6	6.9
	C4	0.5	5.5	1.9	0.9	11	3.8	1.9	23.1	7.7
	F3	0.7	7.7	3.2	1.3	13.7	6.1	2.6	27.4	12.2
	F4	0.8	8.4	3.4	1.4	14.6	6.2	2.6	30.9	12.5

The range of minimum and maximum currents predicted at the ROIs was between 0.5 and 44.8 μA/cm^2^. There was as much as a 10-fold difference among voxels within the spherical ROIs that reflects the complex resistivity within the cortical structures. The range of mean current density values was between 1.7 μA/cm^2^ for the females with 0.5 mA stimulation current at C3 to a high of 17.9 μA/cm^2^ for the males with a 2 mA stimulation current at C3.

The relationship between stimulation current and current density in the ROIs was analyzed in a stepwise manner in order to determine contributions of stimulation intensity, electrode size, and gender to the modeled current density in brain.

**Figure 3:**
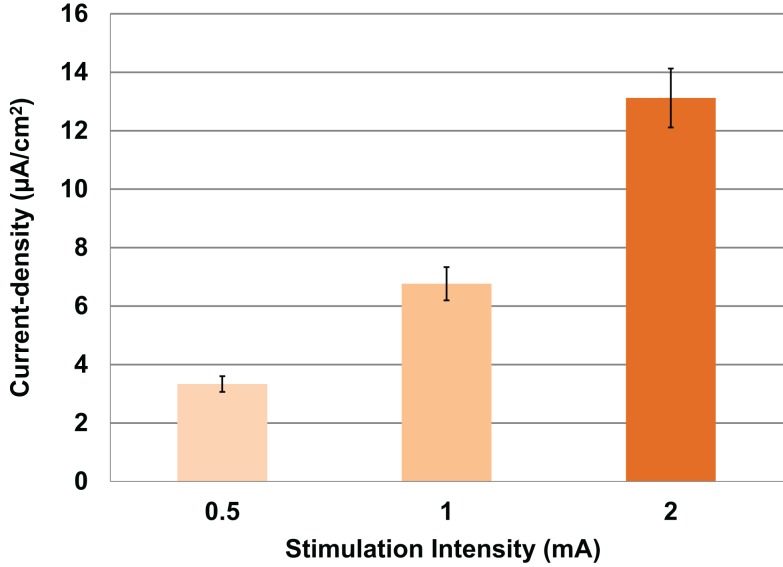
**Above is a graph of the increase in mean current densities for all subjects, electrodes and locations at 0.5, 1, and 2 mA input currents**. Error bars represent ±standard error of the mean.

First, modeled current density was averaged over the four ROIs per subject for both electrode sizes and for combined males and females. Thus, independent of gender, electrode location, and electrode size, modeled current density increased correspondingly to the stimulation intensity (Figure 3). Thus, with each doubling of stimulation intensity, our model predicted significant doubling of current density in the ROIs [*F*(2,69) = 52.3, *P* < 0.001].

**Figure 4:**
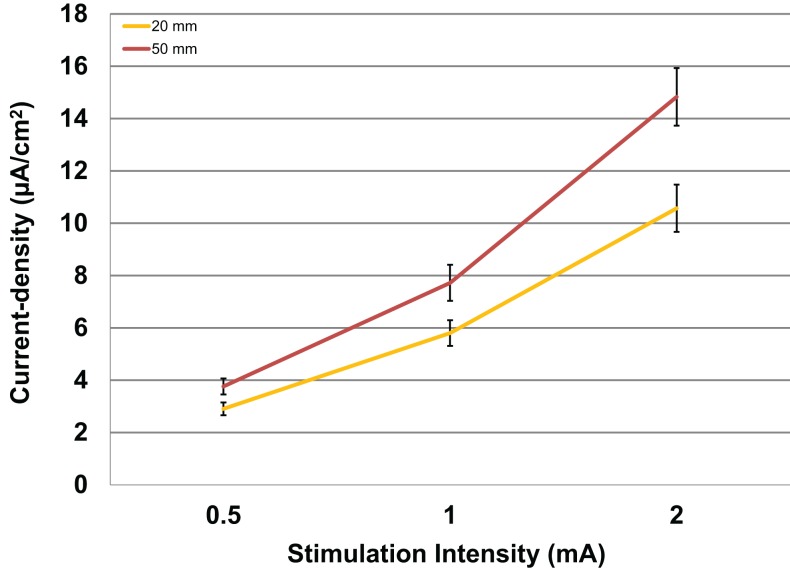
**Above are the mean current densities for the 50 mm × 45 mm electrodes and the 20 mm round electrodes at the three current intensities 0.5, 1, and 2 mA**. Error bars represent ±standard error of the mean.

Modeled current density was next analyzed for differences in electrode size by averaging values over the four ROIs per subject with male and female subjects combined. The relationship between the amount of input stimulation current and the mean current density was nearly linear for both the 20 and 50 mm electrodes (Figure 4). The 50 mm electrodes produced significantly higher current densities than the 20 mm electrodes [*F*(1,46) = 7.138, *P* = 0.010] across all stimulation intensities.

**Figure 5:**
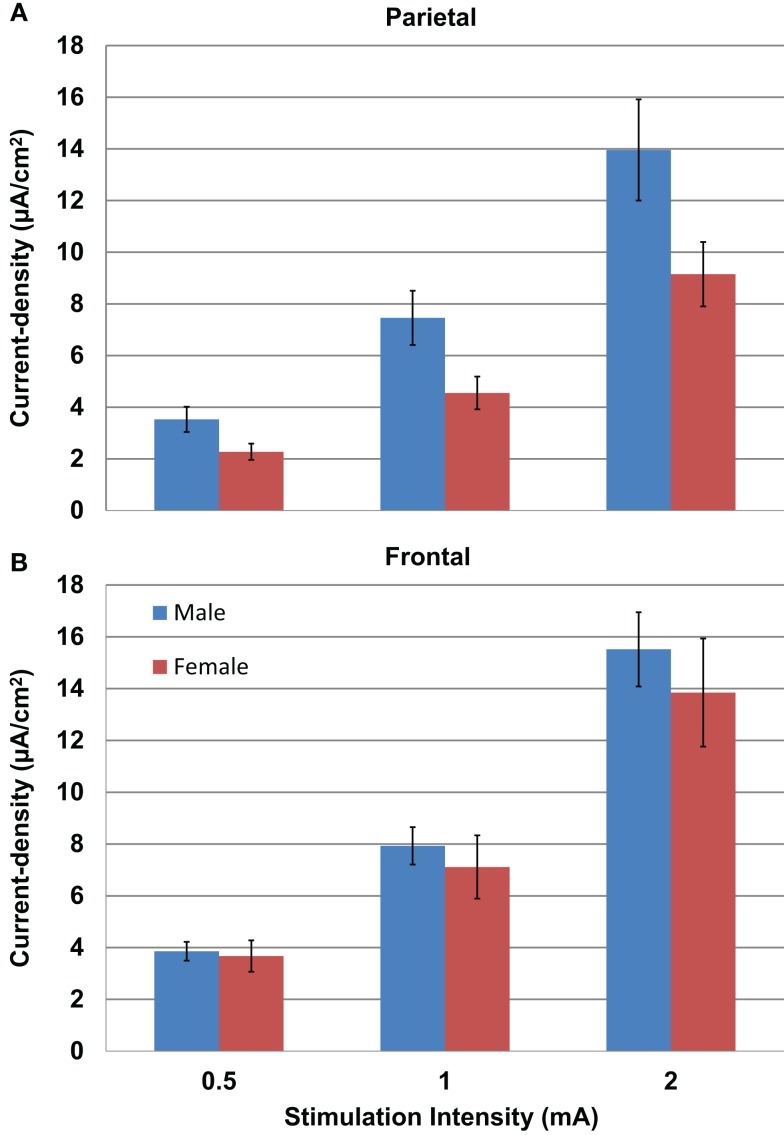
**Above are graphs of the mean current densities for the males (blue) and females (red) at the parietal locations [(A) top] and the frontal locations [(B) bottom]**. Error bars represent ±standard error of the mean.

Modeled current density was next analyzed for differences in gender in parietal (Figure 5A) and frontal stimulation (Figure 5B) sites averaged across electrode size and hemisphere. Males received significantly higher modeled current density than females after parietal stimulation [*F*(1,22) = 4.853, *P* = 0.04]. There was no difference between males and females with frontal stimulation [*F*(1,22) = 0.333, *P* = 0.57].

**Figure 6:**
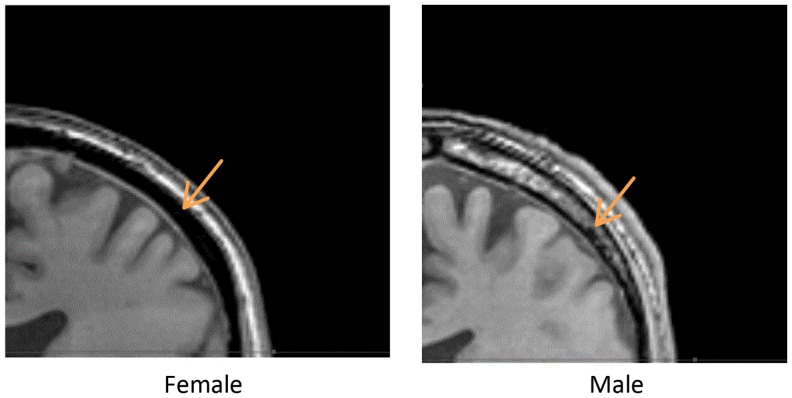
**Above are two parietal coronal MRI sections through male (right) and female (left) heads**. The arrows point to the cancelous bone region.

**Figure 7:**
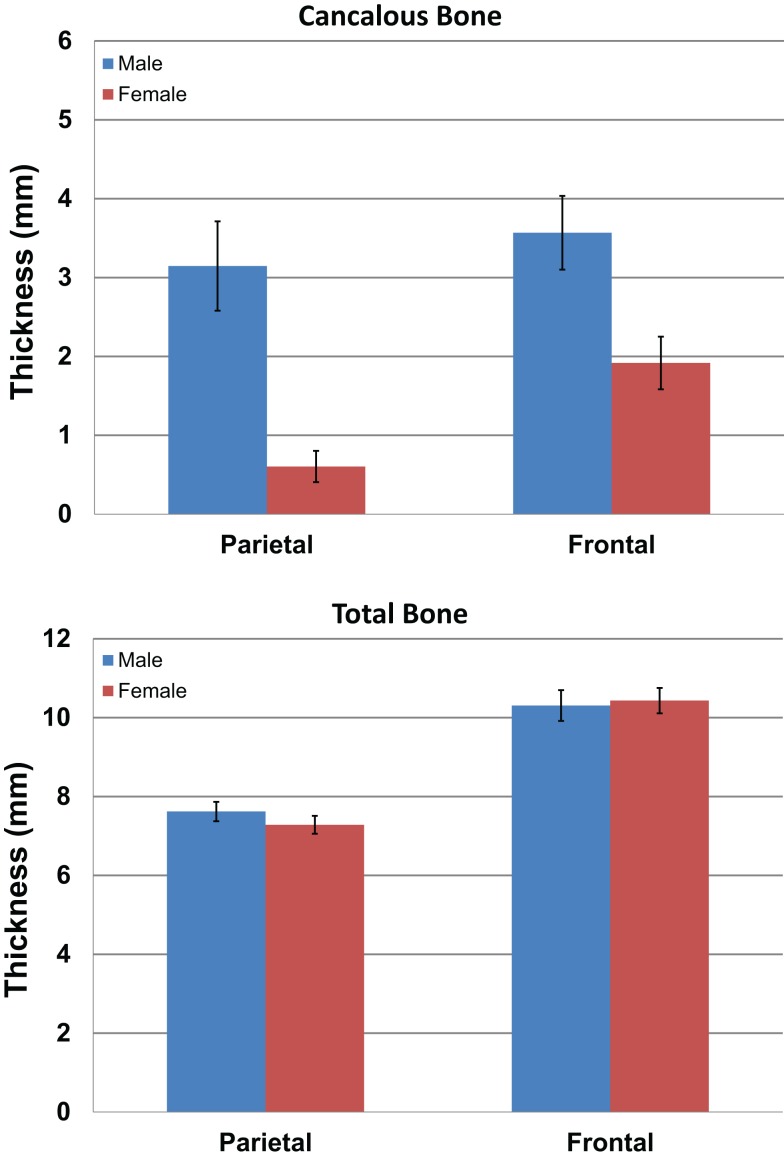
**The graphs show the mean measurements in millimeters of the cancelous bone in the parietal and frontal regions by gender (blue male red female)**. The upper graph is for cancelous bone thickness and the lower graph is total bone thickness. Error bars represent ±standard error of the mean.

We measured the distance between the electrodes and the cortical surface, the thickness of the bone under the electrode, and the thickness of the cancelous bone using the MRI scans. No gender differences were found in the distance between the scalp surface and the cortex at the parietal bone (female mean = 15.03 mm; male mean = 15.08 mm) nor in the frontal bone thickness (female mean = 10.5; male mean = 10.1). No gender differences were found in the total parietal bone thickness (female mean = 7.34 mm; male mean = 7.62 mm). A highly significant difference was found when cancelous parietal bone thickness was compared by gender (Figure 7). The women had denser cortical bone with very little cancelous bone (Figures 6 and 7). Males had significantly thicker cancelous bone in the parietal region [*t*(22) = 4.245, *P* < 0.001] and in the frontal region [*t*(22) = 2.875, *P* = 0.009].

## Discussion

Knowledge of the levels of current received at a target during electrical stimulation is a basic element for the objective evaluation of outcomes. The skull is a high resistance and anatomically complex organ with multiple bone thicknesses and densities that impact current flow. This study suggests that when planning tCS treatments consideration of the regional differences in bone and tissue resistivity should be considered. The cortical targets directly under the stimulating electrodes are receiving a wide range of current in the normal subject population. The large disparity in current densities that we have observed is likely to account for a significant portion of the variation seen in reported patient outcomes. Because of its high resistivity, the bone under the scalp is the most important factor for determining the amount of current received at the targeted cortex. The gender differences in this study were unanticipated. The large differences in parietal current density values were particularly significant. The male subjects received approximately 45% greater current to the cortex than female subjects. Given the large number of investigators using tCS, a more a detailed normative study of bone density, gender, and development should be undertaken. Such a study should examine a larger number of electrode locations and skull regions to provide a detailed map of skull resistivity. There may be other factors that influence skull density and resistivity such as chronic alcohol or drug abuse, smoking, handedness, etc.

Studies that have previously attributed sex differences in tCS results to differences in neural processing, neural plasticity, or hormonal conditions (30, 31) may need to be reconsidered as those differences may have been due to differences in bone density. Studies that have used subjects of both genders may find results more meaningful if the results are analyzed by gender.

The data in this study raise some additional questions. An individual three-dimensional MRI or other analysis of cranial bone is clearly desirable to plan for optimal stimulus intensity, but usually such data is unavailable, and when available it may add significant cost to tCS studies. Should adjustments in stimulation levels be made to compensate for gender and electrode locations on the head? If such a strategy is adopted, exposing women to higher current densities may pose problems with scalp sensations and controlling for placebo effects. Should men receive lower dosages of current than they have been receiving past? In studies where women had a successful outcome, but males did not were the male current levels too high? Recently, there has been some recognition of the need to model stimulation in children (32, 33). These studies may provide us an idea of when gender differences appear.

### Limitations of the study

We did not analyze the effect of distance between electrodes, but decreasing the inter electrode distance reduces the resistance and increases the shunting through the skin. Smaller heads move electrodes closer together and lead to increased shunting. This can be compensated for to some degree by adjusting the stimulation intensity to compensate for shunting through the skin.

The MRI based modeling used in this study was previously validated with 25 surface measurements from the heads of each of three individuals (8), but validation of the results using intracranial measurements have not yet been completed. It is possible that absolute values may need to be adjusted when internal tissue validation is available, but relative values are not likely to change.

The current density values modeled for each spherical 1 cm diameter ROI was the mean of 524 mm^3^ voxels recorded by the MRI. There was considerable variation of current densities between the voxels within that sphere and thus the predicted stimulation by the modeling of individual voxel-sized micro environments are somewhat heterogeneous. Similarly, the resistance modeled for each voxel is a composite value formed from the multiple nervous tissues (neurons, glia, interstitial space, etc.) within each voxel. Thus, the modeling is limited to the precision of the MRI as improvements are made in the quality of MRI recording the precision of the modeling will also improve.

This report is focused on current density, but voltage is a more appropriate measure when the concern is a stimulus locked neuronal discharge. The data in this study are best applied to direct current and slow wave alternating currents and less applicable to transcranial square wave pulse stimulation or studies of stimulation rates above 150 Hz because impedance rather than simple tissue resistance becomes an issue. The optimal current dosage therapeutic window needed to elicit a response likely varies for AC, DC, and random stimulation and depend on the targets selected, but knowing the range of currents that subjects are receiving should help in beginning to establishing optimal levels.

These simulations may be useful in helping investigators evaluate the results published in the literature and in guiding their selection of stimulation levels for future studies.

This study only sampled four locations using the 10–10 system. Many investigators use the 10–20 system. The locations we have chosen C3, C4, F3, and F4 that are identical in the two systems (26). A replication in a larger sample size is warranted to cover the additional locations are specific to the two systems.

Given the linear nature of the relationship of resistivity to current density, it is reasonable to assume that input currents that differ from what we have modeled can be inferred. However, caution should be applied when stimuli are substantially above or below the sampled range.

There are likely larger differences between individuals than what is represented here. Our 24 subjects do not represent the broad range of cranial differences that is seen in the normal population. Although race was not assessed, all of the subjects in this population appeared to be of European decent. The study does not represent the range of body types seen in the human population. For a recent analysis of the diversity, see Ref. (34–36). Clinical factors like bone density, age, cranial anomalies, tumors, inflammation, etc. are likely to further influence current received.

## Conclusion

Current density dosing levels that are too high or too low may produce poor tCS outcomes for patients. The range of current intensities received at the cortex with tCS varies with stimulus intensity, electrode size, scalp location, and subject gender. There can be a significant gender difference in the current received when the brain is stimulated transcranially. In general, men receive more current than women when stimulated above parietal bone. Larger electrodes deliver more current than smaller electrodes. Study designs should consider the influence of electrode size, subject gender, scalp location, and other issues of skull density that may influence stimulus intensity. These variables should be factored in when designing or interpreting study outcomes, and may have important consequences in clinical practice, and for patients who receive tCS treatments for neurological or psychiatric disorders.

## Conflict of Interest Statement

Dr. Michael Russell has two patents that are related to this study. The other authors declare that the research was conducted in absence of any additional commercial or financial conflict of interest.
